# Spatial, temporal and spatio-temporal clusters of measles incidence at the county level in Guangxi, China during 2004–2014: flexibly shaped scan statistics

**DOI:** 10.1186/s12879-017-2357-1

**Published:** 2017-04-04

**Authors:** Xianyan Tang, Alan Geater, Edward McNeil, Qiuyun Deng, Aihu Dong, Ge Zhong

**Affiliations:** 1 Department of Epidemiology and Biostatistics, School of Public Health, Guangxi Medical University, Guangxi Zhuang Autonomous Region, China; 2 Epidemiology Unit, Faculty of Medicine, Prince of Songkla University, Hat Yai, Thailand; 3 Guangxi Center for Disease Control and Prevention, Institute of Vaccination, Guangxi Zhuang Autonomous Region, China

**Keywords:** Spatial, Temporal, Spatio-temporal, Irregular cluster, Measles, Scan statistics

## Abstract

**Background:**

Outbreaks of measles re-emerged in Guangxi province during 2013–2014, where measles again became a major public health concern. A better understanding of the patterns of measles cases would help in identifying high-risk areas and periods for optimizing preventive strategies, yet these patterns remain largely unknown. Thus, this study aimed to determine the patterns of measles clusters in space, time and space-time at the county level over the period 2004–2014 in Guangxi.

**Methods:**

Annual data on measles cases and population sizes for each county were obtained from Guangxi CDC and Guangxi Bureau of Statistics, respectively. Epidemic curves and Kulldorff’s temporal scan statistics were used to identify seasonal peaks and high-risk periods. Tango’s flexible scan statistics were implemented to determine irregular spatial clusters. Spatio-temporal clusters in elliptical cylinder shapes were detected by Kulldorff’s scan statistics. Population attributable risk percent (PAR%) of children aged ≤24 months was used to identify regions with a heavy burden of measles.

**Results:**

Seasonal peaks occurred between April and June, and a temporal measles cluster was detected in 2014. Spatial clusters were identified in West, Southwest and North Central Guangxi. Three phases of spatio-temporal clusters with high relative risk were detected: Central Guangxi during 2004–2005, Midwest Guangxi in 2007, and West and Southwest Guangxi during 2013–2014. Regions with high PAR% were mainly clustered in West, Southwest, North and Central Guangxi.

**Conclusions:**

A temporal uptrend of measles incidence existed in Guangxi between 2010 and 2014, while downtrend during 2004–2009. The hotspots shifted from Central to West and Southwest Guangxi, regions overburdened with measles. Thus, intensifying surveillance of timeliness and completeness of routine vaccination and implementing supplementary immunization activities for measles should prioritized in these regions.

## Background

Measles is a highly contagious disease and a leading cause of death among children worldwide [1]. More than 95% of measles deaths have been reported in low-middle income countries and regions with resource-poor health facilities [2]. China, a middle-income country, is one of the member nations in the WHO Western Pacific Region, accounting for approximately 70% of the reported measles cases in this region since 2003 [3]. According to the schedule of expanded program on immunization (EPI) in China, routine measles vaccination covers two doses of measles-containing vaccine (i.e. MCV1, MCV2). Children should be vaccinated with MCV1 at the age of 8 months, and MCV2 should be given to children between the ages of 18 and 24 months. In addition, the current schedule of supplementary immunization activities (SIA) for measles is during September 11–20 per year. Despite the routine two-dose measles vaccination coverage across most regions of China being over 90%, large-scale outbreaks of measles still occurred in Guangxi, Beijing, Zhejiang and Shangdong provinces [4–7].

Prior to 2003, Guangxi was one of the provinces in China with the highest measles incidence [8]. Since 2004, the incidence has dropped below the national average [8]. However, large-scale measles outbreaks re-emerged in Guangxi during 2013–2014 [9]. Therefore, measles again became a major public health concern in Guangxi. To effectively improve the timeliness of routine measles vaccination and to implement supplementary immunization activities for measles in high-risk regions, a better understanding of the spatial, temporal and spatio-temporal patterns of measles is needed. Unfortunately, these patterns remain largely unknown in Guangxi.

Scan statistics, which are used in spatio-temporal analyses, have been widely applied to explore the patterns of disease and detect high-risk diseases clusters, particularly for diseases having limited resources for prevention [10]. However, most previous studies examined regular (i.e. circular or cylindrical shape) diseases clusters, which are unrealistic, since the true shapes of clusters are usually less regular [11–16]. Few studies have explored irregularly shaped diseases clusters, which might more realistically reflect the actual, albeit complicated, patterns of disease [17, 18]. Moreover, most studies have utilized the relative risk (RR) derived from scan statistics to identify high-risk regions [11, 13, 14, 16]. Few studies have measured the population attributable risk percent (PAR%) to evaluate the regional burden of disease [7]. Additionally, there is a paucity of studies on the spatio-temporal patterns of measles, particularly in Guangxi, a region with large-scale measles outbreaks.

Therefore, a spatio-temporal analysis was conducted to identify spatial, temporal and spatio-temporal irregular clusters of measles incidence at the county level in Guangxi between 2004 and 2014, integrating flexibly shaped scan statistics and PAR%. Results of this study may not only help understand the spatio-temporal shift trend of measles in Guangxi, but also facilitate evaluating the geographical inequities in the burden of measles. Moreover, findings may provide essential evidence for implementing supplementary immunization activities for measles and strengthening the surveillance of timeliness and completeness of routine measles vaccination in overburdened regions, as well as other developing countries with a similar context.

## Methods

### Study area

The study site was Guangxi Zhuang Autonomous Region, a mountainous province in Southwest China. Geographically, Guangxi has proximity to the Association of Southeast Asian Nations (ASEAN) member states. It has a subtropical climate, with high humidity and temperature. Guangxi consists of 14 prefectures and 109 counties or urban districts, with an area of 236,700 km^2^ and a population of 46.8 million residents in 2012. In this study, a total of 109 counties or urban districts were included for spatio-temporal analysis.

### Data sources

Yearly data on measles cases for each county between 2004 and 2014 was obtained from the National Notifiable Infectious Disease Surveillance System in Guangxi Center for Disease Control and Prevention. As a class B notifiable disease, all measles cases were laboratory confirmed by at least two experts according to the WHO manual for laboratory diagnosis of measles virus infection [19]. The annual population size and the proportion of the total number of children aged ≤24 months for each county during 2004–2014 was obtained from Guangxi Bureau of Statistics. Based on the population size and the number of notifiable measles cases, the annual incidence (per 100,000 population) was calculated for each county. In addition, a vector map of Guangxi at the county level was collected from Guangxi Bureau of Surveying, Mapping and Geoinformation.

### Temporal, spatial and Spatio-temporal analysis

An epidemic curve of monthly measles cases during January 2004 to December 2014 was drawn to reveal the seasonal peaks, and a trend line was fitted to identify the annual trend in incidence. Purely temporal clusters of high incidence were detected by Kulldorff’s temporal scan statistics based on a discrete Poisson model in SaTScan 9.4.2 [10].

Inverse distance weighted interpolation of annual incidence was calculated in Arc GIS 10.0 to generate smoothed maps of measles incidence [20]. Spatial irregular clusters of high-incidence were tested by Tango’s flexible spatial scan statistics in FleXScan 3.1.2, using a Poisson model with restricted log likelihood ratio (default alpha = 0.2) and 15 census areas as the maximum spatial cluster size [21, 22]. These hotspots were visualized in Arc GIS 10.0.

Kulldorff’s retrospective space-time scan statistics based on the Poisson model and elliptical-cylinder scanning window in SaTScan 9.4.2 was applied to detect irregular spatio-temporal clusters of high-incidence during 2004–2014, which were visualized in three-dimensions in AutoCAD 2010 [10, 18].

Generally, Kulldorff’s retrospective scan statistics are used to detect high-risk clusters in either a purely temporal, purely spatial or jointly spatio-temporal setting, based on a gradual scanning window [10]. The scanning window is an interval for purely temporal scan statistics, and a circle or ellipse for purely spatial scan statistics. For retrospective spatio-temporal scan statistics, the scanning window is a cylindrical or elliptical-cylinder shape with a base corresponding to space and with height corresponding to time. The window moves over space and time scanning for elevated risks inside the window as compared to outside. For each change in space and time in the window, the log likelihood ratio (LLR), a statistic is automatically calculated. The window with the highest maximum LLR is deemed to be the most likely cluster. The *P*-value for clustering is calculated using Monte Carlo simulation.

Relative risk (RR) was determined in the spatio-temporal scan statistics analysis, the estimated risk within the cluster divided by the estimated risk outside the cluster. In this perspective, the reference group for calculating RR is the estimated risk outside the cluster. Mathematically, RR is calculated as the observed cases divided by the expected cases within the cluster divided by the observed cases divided by the expected cases outside the cluster.

The elliptical-cylinder was the preferred shape for use during scanning to detect irregular clusters of space-time in this study. The maximum spatial cluster size was set to 15% of the population at risk to avoid falsely absorbing neighborhood regions into an unrealistic cluster, and the maximum temporal cluster size was set to the default 50% of the scan timeframe [23]. For statistical power evaluation, the number of Monte Carlo replications was set to 999. Temporal trend adjustment was conducted by log linear model with automatically calculated trend, and spatial trends were adjusted by nonparametric statistics with spatial stratified randomization. Clusters were reported without geographical overlap.

### Population attributable risk percent

From a public health perspective, the population attributable risk percent (PAR%) reflects the fraction of all cases in the population attributable to being in that cluster [24]. For disease burden analysis, we calculated PAR% by county to estimate how much measles could be avoided if routine two-dose measles vaccination was timely and completely delivered to population aged ≤24 months. The formula for PAR% is listed as follows [7]:

$$ PAR\%=\frac{P_E\left( RR-1\right)}{P_E\left( RR-1\right)+1}\times 100 $$

where, RR is the relative risk of measles in each county, which was derived from the results of spatio-temporal scan statistics in SaTScan software, and *P*_E_ is the proportion of the population aged ≤24 months in each county. As *P*_E_ can not be reduced, a reduction in PAR% can be achieved only by reducing the RR. This requires an increased coverage of timely and complete measles vaccination in the county. Namely, the routine two-dose measles vaccines should be delivered timely to children aged ≤24 months in the county, according to the national immunization schedule of China [25].

### Linear directional mean

The mean center of measles incidence is the geographic average location of a set of points. In this study, the mean centers of the locations of the counties having measles case were measured. And the mean centers denote the area and extent of the incidence of measles cases. The linear directional mean was conducted in Arc GIS 10.0 to measure the directional trend of measles transmission in Guangxi, using the annual mean center movements of measles incidence during 2004–2014 [26].

### Ethical considerations

This study was approved by the Ethics Committees of Prince of Songkla University and Guangxi Medical University. Dataset of measles cases was approved by Guangxi Center for Disease Control and Prevention.

## Results

### Purely temporal clusters of measles

**Fig. 1:**
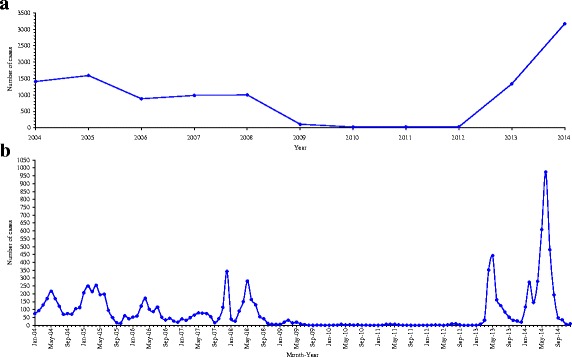
Annual trend in measles incidence during 2004–2014 and epidemic curve of monthly measles cases in Guangxi, between January 2004 and December 2014. **a** is for trend line, and **b** is for epidemic curve

Figure 1 shows the monthly distribution of measles cases between 2004 and 2014 and the temporal trend in measles incidence. Prior to 2009, seasonal peaks occurred predominantly during April to June in Guangxi with one unseasonal peak occurring in December 2007. No further peaks occurred until May 2013. Overall, a two-piecewise temporal trend of measles incidence was observed during the whole 11-year period, i.e. downtrend during 2004–2009 and uptrend during 2010–2014. And a purely temporal cluster of very high incidence was detected in 2014 (LLR = 1570.73, RR = 3.76, *P* = 0.001).

### Purely spatial irregular clusters of measles

**Fig. 2:**
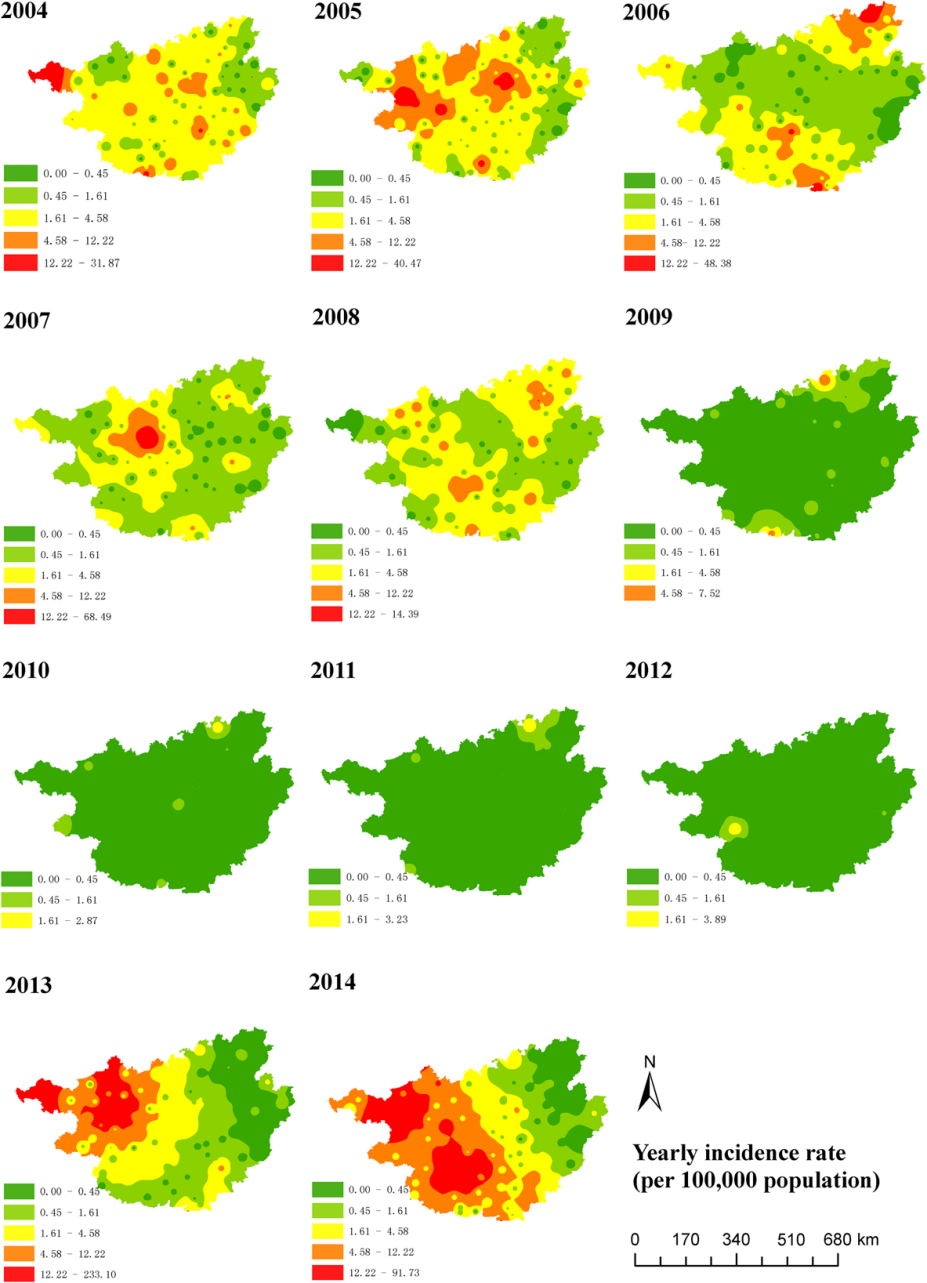
Spatial interpolation of measles incidence between 2004 and 2014, Guangxi, China

**Fig. 3:**
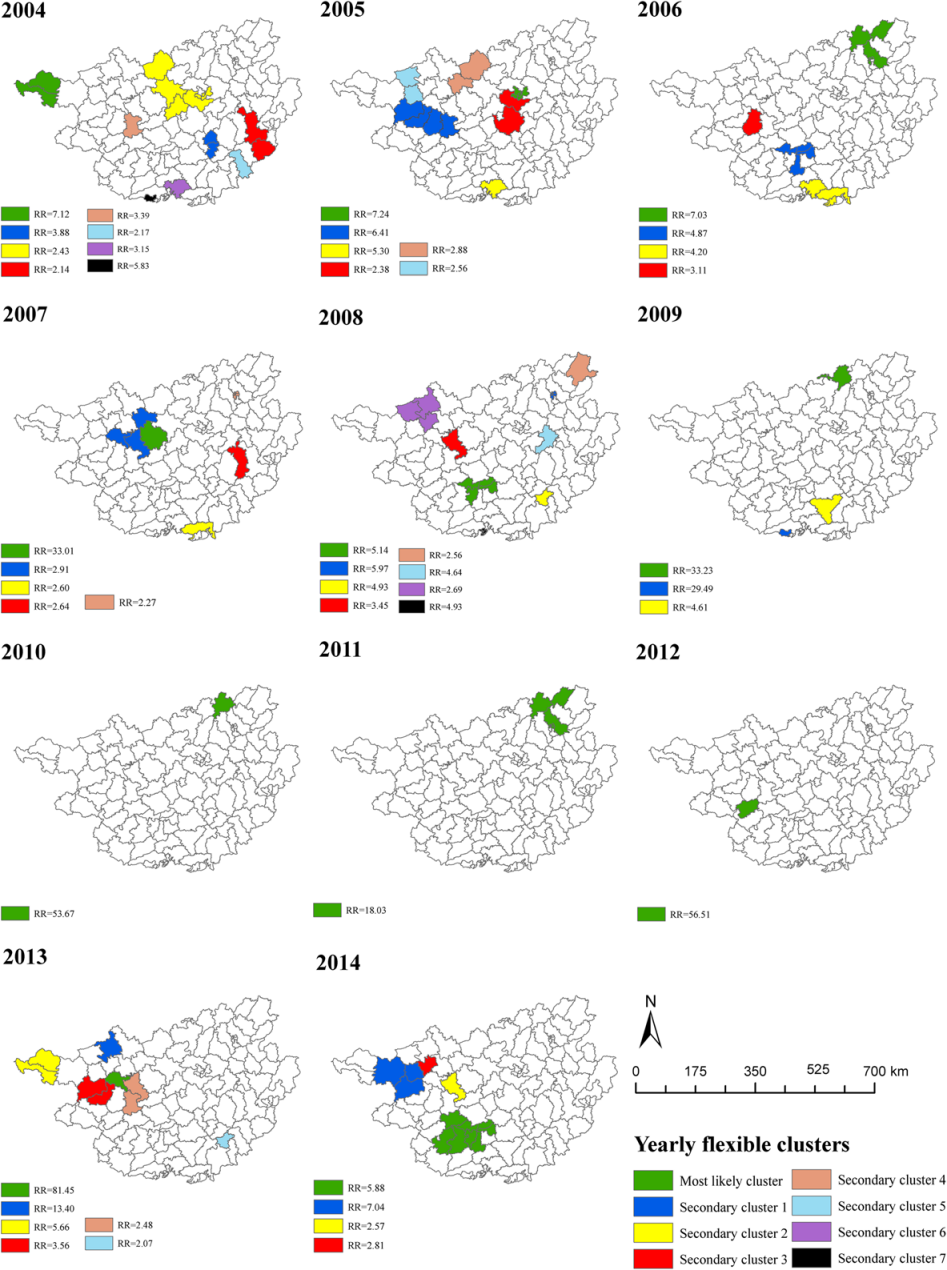
Spatial irregular clusters of measles incidence between 2004 and 2014, Guangxi, China

Figure 2 shows the spatial interpolation of annual incidence between 2004 and 2014. In general, West, Southwest and North Central Guangxi were the hotspots of measles. Table 1 presents the spatial clusters of high incidence, which are visualized in Fig. 3. The spatial pattern of clustering was consistent with that of incidence interpolation shown in Fig. 2.

**Table 1:** Spatial irregular clusters of annual measles incidence between 2004 and 2014

Year	Type of cluster	No. of counties in cluster	Radius (km)	LLR	RR	*P*-value^*^
2004	Most likely cluster	2	39.17	114.21	7.12	0.001
	Secondary cluster 1	8	164.99	104.56	2.76	0.001
	Secondary cluster 2	6	187.76	38.18	2.06	0.001
	Secondary cluster 3	4	113.48	26.68	1.75	0.001
	Secondary cluster 4	3	81.03	24.37	2.66	0.001
2005	Most likely cluster	4	26.18	381.42	7.24	0.001
	Secondary cluster 1	4	117.62	355.75	6.41	0.001
	Secondary cluster 2	1	0.00^a^	85.96	5.30	0.001
	Secondary cluster 3	2	61.69	35.73	2.38	0.001
	Secondary cluster 4	2	64.71	28.58	2.88	0.001
	Secondary cluster 5	2	53.88	9.99	2.56	0.033
2006	Most likely cluster	6	100.54	191.49	7.03	0.001
	Secondary cluster 1	6	132.81	137.54	3.99	0.001
	Secondary cluster 2	2	12.85	91.45	8.48	0.001
	Secondary cluster 3	5	179.29	11.43	1.93	0.005
2007	Most likely cluster	1	0.00^a^	1107.94	33.01	0.001
	Secondary cluster 1	3	87.64	25.58	2.91	0.001
	Secondary cluster 2	2	32.53	23.32	2.60	0.001
	Secondary cluster 3	1	0.00^a^	22.49	2.64	0.001
	Secondary cluster 4	4	14.26	9.88	2.27	0.046
2008	Most likely cluster	4	59.44	207.67	5.14	0.001
	Secondary cluster 1	4	14.26	96.97	5.97	0.001
	Secondary cluster 2	1	0.00^a^	87.52	4.93	0.001
	Secondary cluster 3	4	153.81	19.88	2.52	0.001
	Secondary cluster 4	1	0.00^a^	12.49	2.56	0.002
	Secondary cluster 5	1	0.00^a^	10.57	4.64	0.018
	Secondary cluster 6	1	0.00^a^	9.62	4.93	0.050
2009	Most likely cluster	1	0.00^a^	66.28	33.23	0.001
	Secondary cluster 1	5	136.07	20.64	4.62	0.001
2010	Most likely cluster	1	0.00^a^	15.51	53.67	0.002
2011	Most likely cluster	6	92.81	28.95	18.03	0.001
2012	Most likely cluster	1	0.00^a^	42.68	56.51	0.001
2013	Most likely cluster	1	0.00^a^	1924.26	81.45	0.001
	Secondary cluster 1	8	205.23	163.35	4.14	0.001
	Secondary cluster 2	2	49.60	18.22	2.48	0.001
	Secondary cluster 3	1	0.00^a^	13.63	2.07	0.001
2014	Most likely cluster	8	127.25	2006.30	5.88	0.001
	Secondary cluster 1	3	71.96	431.92	7.04	0.001
	Secondary cluster 2	5	153.81	24.99	1.88	0.001

*Tango’s flexible scan statistics

^a^Zero-radius means only one county in the cluster

### Spatio-temporal irregular clusters of measles

**Fig. 4:**
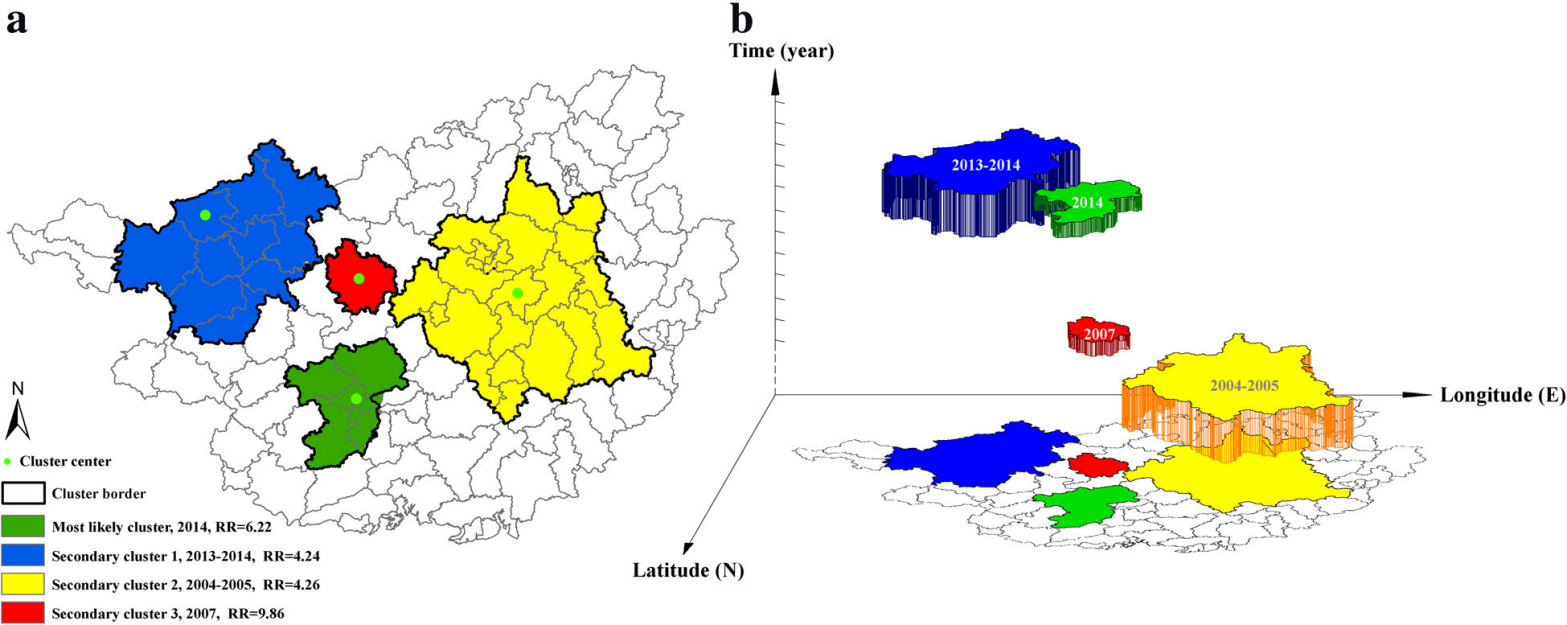
Spatio-temporal irregular clusters of measles incidence in Guangxi, between 2004 and 2014. **a** and **b** are the visualizations in two-dimension and three-dimension, respectively

**Fig. 5:**
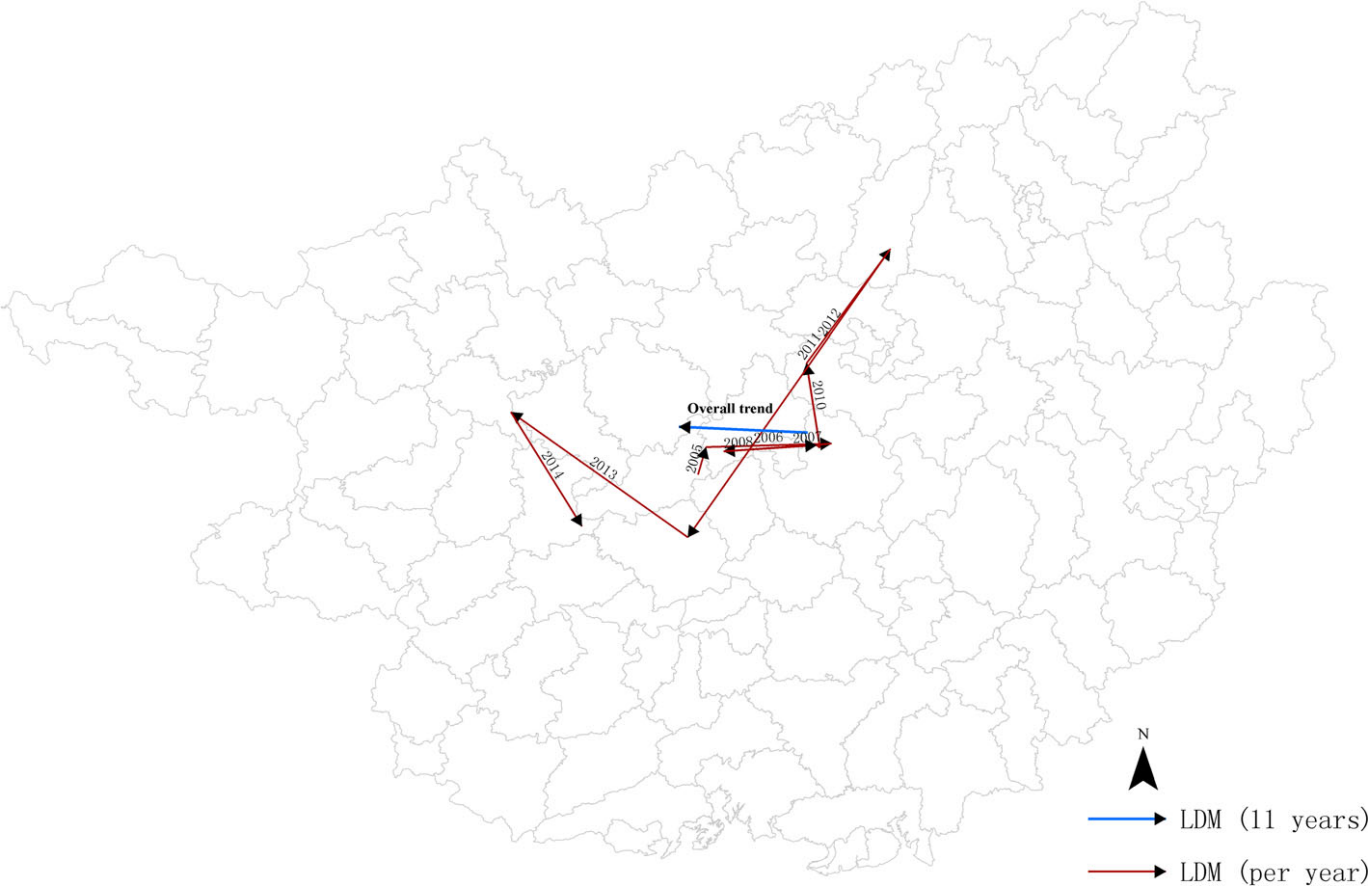
Linear directional mean (LDM) during 2004–2014

Table 2 summarizes the spatio-temporal clusters of high incidence between 2004 and 2014, which are visualized in Fig. 4. In general, there were three distinct phases of space-time clusters. Specifically, spatio-temporal clusters were identified in Central Guangxi during 2004–2005 (Phase 1), Midwest Guangxi in 2007 (Phase 2), and West and Southwest Guangxi during 2013–2014 (Phase 3). Moreover, a spatio-temporal trend of measles was observed, shifting from Central to West and Southwest Guangxi, which was revealed by linear directional mean as well (Fig. 5).

**Table 2:** Spatio-temporal irregular clusters of annual measles incidence during 2004–2014

Type of cluster	Cluster period	Cluster center	No. of counties	LLR	RR	*P*-value^*^
Most likely cluster	2014	108.08 E, 22.92 N	5	1042.05	6.22	0.001
Secondary cluster 1	2013–2014	106.51 E, 24.83 N	10	764.37	4.24	0.001
Secondary cluster 2	2004–2005	109.76 E, 24.02 N	23	637.35	4.26	0.001
Secondary cluster 3	2007	108.11 E, 24.17 N	1	552.47	9.86	0.001

^*^Kulldorff’s space-time scan statistics based on Poisson model and elliptical-cylinder scanning window

### Patterns of RR and PAR%

**Fig. 6:**
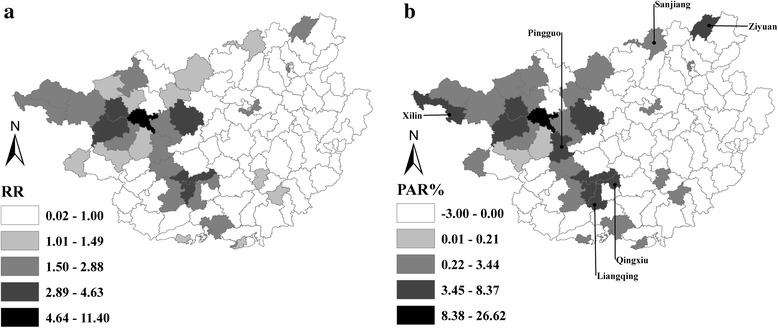
Spatial patterns of relative risk (RR) and population attributable risk percent (PAR%) among children aged ≤24 months in Guangxi, 2004–2014. **a** is for RR, and **b** is for PAR%

Figure 6 reveals the spatial patterns of RR and PAR% among children aged ≤24 months in Guangxi, during 2004–2014. In general, the pattern of RR mostly coincided with that of PAR%. Regions with high RR and PAR% were mainly clustered in West, Southwest, and North Central Guangxi. Despite the geographical similarity between the two indicators, heterogeneities in some regions were seen in Xilin, Pingguo, Liangqing, Qingxiu, Ziyuan and Sanjiang counties, with a low or moderate level of RR but high level of PAR% evident. Thus, a low RR might obscure the fact that some low-risk regions had a heavy burden of measles.

## Discussion

The present study is the first attempt to detect irregular clusters of space, time, and space-time, combining flexibly shaped scan statistics and population attributable risk percent. The findings revealed that a temporal uptrend of measles incidence existed in Guangxi between 2010 and 2014, spatio-temporal clusters of measles had shifted from Central to West and Southwest Guangxi, and regions with high RR and PAR% were mainly clustered in West, Southwest and North Central Guangxi. These findings not only deepen our understanding of the spatio-temporal patterns of measles in Guangxi, but also assist in identifying regions with high risk and heavy burden of measles, where intensifying surveillance of timeliness and completeness of routine measles vaccination and implementing supplementary immunization activities for measles should be prioritized.

Yellow card alerting system, an innovative vaccination intervention way, works as a core tool for policy intervention to classify counties alerted by a yellow card into EPI blacklist [27]. Since the yellow card alerting system, routine two-dose measles vaccination program and supplementary immunization activities for measles were implemented in Guangxi, the measles incidence has remained at a low level [27]. However, large-scale outbreaks of measles re-emerged in Guangxi during 2013–2014, possibly attributed to the accumulation effect of a fairly large number of susceptible children in the last decade, which ultimately weakened the herd immunity against measles [28]. The seasonal peaks of measles predominantly occurred between April and June, in addition to a minor peak in December 2007. The minor seasonal peak could be due to the abnormally high temperatures in winter during that year, which created ideal conditions for the spread of measles [29].

Overall, purely spatial clusters of measles constantly emerged in West, Southwest, North and Central Guangxi, though the endemic areas of measles had considerably shrunk after the implementation of the expanded program on immunization. With parents migrating to urban areas for employment opportunities, numerous children were left behind in rural areas, particularly in impoverished and remote areas, where the travel time to vaccination clinics are long and the timeliness and completeness of measles vaccination was low [9]. This might be the reason why the clusters were located in West and North Guangxi, regions with high labour exports. Simultaneously, many migrant children in urban or semi-urban areas did not receive measles vaccination and thus became susceptible to measles [30]. In this light, it was not surprising that clusters of measles occurred in labour-importing regions as well, i.e. Liuzhou in Central and Nanning in Southwest Guangxi.

Regarding the populations vulnerable to non-immunization, previous studies revealed that both left-behind children and migrant children were the disadvantaged groups in China and suffered from inequities in timely vaccination service [9, 30, 31]. Furthermore, these children have brought great challenges to the elimination of measles in China. Thus, the shift of space-time clusters from Central to West and Southwest Guangxi might be partly because of the dynamic changes in demographic structure among children in the above regions.

Although a similar spatial pattern of measles was identified in terms of RR and PAR%, heterogeneity still existed in Xilin, Pingguo, Liangqing, Qingxiu, Ziyuan and Sanjiang counties. These regions had low or mild levels of RR but a high level of PAR%, probably because of the high-density of children aged ≤24 months living there. In China, two doses of measles vaccine should be routinely delivered to children within 24 months after birth [25]. Given the advantage of PAR% in evaluating the public health significance of preventive measures, the burden of measles would be reduced sharply if routine two-dose measles vaccination and supplementary immunization activities for measles were delivered on time to the age-appropriate children in regions with high PAR% [7]. Therefore, combining PAR% with scan statistics would be a novel approach in the analysis of disease clusters.

The shape of the scanning window is critical for scan statistics and affects the ability to detect clusters of disease accurately and sensitively [32]. So far, numerous studies have applied a circular or cylindrical window to detect regular and compact clusters [11–16]. However, these windows might fail to reflect the true shapes of clusters, due to the fact that clusters, in reality, are irregular, eccentric and complex [22, 23, 33]. Thus, regularly shaped clusters should be interpreted with caution. To accurately depict the shapes of non-compact clusters and to avoid falsely absorbing neighbouring regions where the actual hazard is not elevated into clusters, we preferred flexibly shaped scan statistics to detect irregular clusters of measles. This is another novelty of the present study.

This study has some limitations. First, the shapes of spatio-temporal clusters were somewhat inflexible. Although the elliptical-cylinder scanning window is more sensitive to clusters that are eccentric, non-compact and noncontiguous in shape than the cylindrical window, it is impossible to completely reflect the true shape of an irregular cluster. Second, the modifiable areal unit problem was inevitable, comprising a zoning effect and a scale effect [34]. On one hand, the adjustment of administrative district at county-level might lead to a zoning effect, which would slightly affect the spatio-temporal patterns of measles. On the other hand, since data at the town-level were not available, the county-level was deemed as the spatial scale to detect clusters. The aggregated data at county-level might obscure the clusters detected at town-level, resulting in the so called ecological fallacy, which is a sub-problem of scale effect. Third, setting the maximum spatial cluster size of the scanning window is somewhat arbitrary, though we preferred 15% of population at risk recommended by other studies [22, 23]. Fourth, the accuracy of the flexibly shaped scan statistics was not evaluated in this study [33, 35, 36]. Fifth, PAR% was underestimated, as we used the proportion of population aged ≤24 months as the percent of exposure to measles, which might not exactly reflect the level of exposure to measles in the whole population. Lastly, factors underlying the spatio-temporal shift trend of measles were not explored quantitatively. Further studies will be conducted to determine the role of socio-economic and demographic factors, geographical barriers and vaccination provider-related determinants in driving the transmission dynamics.

Despite these limitations, our findings have important implications for vaccination policymakers, vaccination practitioners and public health authorities to take essential measures against outbreaks of measles in Guangxi. Given regions with high PAR%, which were mainly clustered in West, Southwest and North Central Guangxi, supplementary immunization activities for measles should be given priority in these regions. Given the shift of high-risk clusters of measles to West and Southwest Guangxi, a prospective real-time measles surveillance system based on prospective scan statistics should be established in these areas to improve the timeliness and completeness of routine measles vaccination among age-appropriate children. In view of the increasing temporal trend of measles incidence, more measles-related health resources should be allocated in Guangxi than in previous years, particularly in high-risk and heavy-burden regions, and more attention should be paid to develop an early-warning system of measles outbreaks.

## Conclusion

This exploratory study integrated flexibly shaped scan statistics and PAR% to detect spatial, temporal and spatio-temporal measles clusters in irregular shapes. The findings revealed that there was a temporal uptrend in measles incidence in Guangxi during 2010–2014, irregular clusters of space-time had shifted from Central to West and Southwest Guangxi, and regions of high risk and heavy burden of measles were mostly concentrated in the West, Southwest and North Central regions of Guangxi. These findings strongly suggest that measures targeting these regions should be developed to prevent future measles outbreaks, and reduce geographical inequities in the burden of measles. Moreover, combination of flexibly shaped scan statistics and PAR% is a novel approach to improve disease cluster detection, which would be widely used for other similar studies.

## Abbreviations

ASEAN: Association of Southeast Asian Nations
CDC: Center for Disease Control and Prevention
GIS: Geographic Information Systems
LLR: Log Likelihood Ratio
PAR%: Population Attributable Risk Percent
RR: Relative Risk
WHO: World Health Organization.
